# Coreografia povoada: cartografia histórica, política e ambiente na disputa da serra da Ibiapaba entre Piauí e Ceará

**DOI:** 10.1590/S0104-59702026000100011

**Published:** 2026-06-29

**Authors:** Luís Carlos Albano Duarte Sousa

**Affiliations:** i Doutorando, Programa de Pós-graduação em História/Universidade Federal de Minas Gerais. Belo Horizonte – MG – Brasil. luis-albano@hotmail.com

**Keywords:** Cartografia histórica, Ceará e Piauí, Jurisdição, Litígio, Missões jesuíticas, Historical cartography, Ceará and Piauí, Jurisdiction, Litigation, Jesuit missions

## Abstract

Este artigo explora os usos da cartografia histórica na disputa territorial entre Piauí e Ceará, ajuizada em ação cível originária n.1.831/2011 no Supremo Tribunal Federal. Propomos um exercício analítico a partir de mapas históricos dos séculos XVII a XIX, no intento de perceber as continuidades e transformações nos modos de representar a região. Contrastamos esse exercício com documentação do Arquivo Histórico Ultramarino e com o Relatório Técnico do Exército Brasileiro sobre a disputa. Com isso, buscamos relacionar a agência do ambiente, influências políticas e representações das zonas litigantes, demonstrando como a cartografia permanece utilizada como “espelho do mundo”.

## Abstract (en)

This article explores the uses of historical cartography in the territorial dispute between the states of Piauí and Ceará in a civil suit (1.831/2011) filed in Brazil’s Supreme Court. We base our analysis on historical maps from the seventeenth to the nineteenth centuries in order to understand continuities and transformations in how this region is represented. This analysis is then contrasted with documentation from the Overseas Historical Archive and the Brazilian Army’s Technical Report on the dispute in order to relate the active role played by the environment, political influences with representations of the zones involved in the dispute, showing how cartography still serves to “reflect the world.”

E a ilha desconhecida, perguntou o homem do leme, A ilha desconhecida é coisa que não existe, não passa de uma ideia da tua cabeça, os geógrafos do rei foram ver nos mapas e declararam que ilhas por conhecer é coisa que se acabou desde há muito tempo.

José Saramago, *O conto da ilha desconhecida*.

## “Maior caráter informacional que métrico”

O litígio entre os estados brasileiros do Ceará e Piauí pela fronteira territorial leva-nos a uma importante reflexão histórica sobre a cartografia e seus usos dentro da ação cível originária n.1.831, ajuizada pelo estado do Piauí, em 2011, junto ao Supremo Tribunal Federal (STF). Depreende-se daí um esforço em acompanhar as movimentações do processo, contrastando-as com uma historiografia preocupada com as disputas políticas, sociais, econômicas e culturais na demarcação de fronteiras e nas suas representações cartográficas. Dado que a ação remete ainda ao século XVIII, não objetivamos aqui um estudo totalizante de todos os âmbitos suscitados por essas disputas, mas reforçamos que todo desenho de fronteira pode e deve ser historicizado para não parecer isento ou gratuito, levando em conta as intenções coevas.

A disputa territorial tem como marco legal o decreto imperial n.3.012, datado de 22 de outubro de 1880, que altera a linha divisória das, até então, províncias do Ceará e Piauí, concedendo a esta as vertentes ocidentais da Serra Grande, ou serra da Ibiapaba – centro da controvérsia. Além disso, foi alegado o Convênio Arbitral assinado pelas duas partes em 1º de julho de 1920, na Conferência de Limites Interestaduais, na qual a República se comprometia a enviar engenheiros competentes, a fim de realizar um levantamento topográfico e definir os ajustes dos limites territoriais entre os dois estados. A questão permaneceu em aberto até 2011, com a abertura do processo no STF.

O ministro Dias Toffoli acolheu a ação do estado do Piauí e incluiu também a União como litisconsorte passivo necessário. Não obtivemos acesso a todas as peças do processo na íntegra, mas as informações públicas disponíveis indicam a sugestão, por parte da União, de submeter a questão à Câmara de Conciliação e Arbitragem da Administração Federal, proposta que foi aceita pelas duas partes, como indica o despacho de 18 de abril de 2012. No ano seguinte, consta como frustrada a tentativa conciliatória, e, em 2016, o Exército Brasileiro foi intimado a se manifestar sobre a possibilidade de uma perícia técnica. Já em 2018, a relatoria do processo passou para a ministra Cármen Lúcia, que, apenas em 2023, publicou uma decisão monocrática em que o Exército informava o período de agosto a outubro do mesmo ano, para que militares do Serviço Geográfico do Exército Brasileiro executassem o trabalho de campo e elaborassem um relatório sobre o qual trataremos adiante.

Em 2024, o *site* UOL veiculou uma matéria intitulada “STF: mapa de 1840 achado em Londres vira ‘prova’ do PI em litígio contra CE” (Madeiro, 1 jun. 2024). Não foi a primeira vez que a cartografia histórica foi suscitada como argumento na questão, mas o objetivo da utilização desse tema e dessa notícia encontra razão no importante debate sobre história pública e o consequente uso da história em espaços mais amplos que os disciplinares, tais como recursos jornalísticos e de formação de opinião popular. Entendemos como história pública aquilo que Jurandir Malerba (2014) conceitua como os esforços dos historiadores e historiadoras, e de seus métodos, empreendidos fora do ambiente da academia, atentos à sua “audiência” e à influência sobre aqueles a quem o conteúdo histórico é dirigido. Portanto, é dever também nosso ocupar os mais diversos lugares onde a opinião histórica é formada, e faz-se imperativo confrontarmos os objetivos implícitos nos usos da história para finalidades atuais.

Provocado pelo STF, o Exército Brasileiro, por meio da Diretoria de Serviço Geográfico, elaborou o *Relatório técnico referente à Ação Cível Originária n.1.831* (Brasil, 2024), com 317 páginas mais anexos. Esse trabalho orientou-se por questionamentos das duas partes, tais como: seria possível dividir equitativamente os territórios em litígio? Assim procedendo, existem meios de evitar prejuízo territorial para os entes federados? Há como traçar os limites sem prejuízos culturais às populações? O que fazer com os equipamentos públicos localizados nas zonas de litígio? No último recenseamento, as populações dessas zonas se consideram pertencentes ao Ceará ou ao Piauí? O objetivo do relatório é fornecer dados atuais para deliberação e, como ambas as partes utilizaram mapas históricos para argumentação, fazer uma retrospectiva para análise de aproximadamente noventa mapas, sendo 18 deles com datação entre 1760 e 1892.

Temos, portanto, dois usos de mapas históricos – um público, outro técnico – que impulsionam a nossa análise. Até o século XIX, os mapas – em sua quase totalidade restritos ao patrocínio dos príncipes e utilizados como armas do imperialismo, conforme Harley (2009) – acomodavam espelhos do mundo, algo explícito no excerto de Saramago que nos serve de mote. Especialmente no período colonial, obedeciam a um esquema comum ao que aconteceu nos sertões do Brasil, qual seja: reconhecimento, coleta de informações gerais e inserção da “civilização” com consequente exploração. A história dos mapas está intimamente ligada à criação dos Estados modernos (inclusive internamente) que permaneceram como “mandatários da atividade cartográfica” (Harley, 2009, p.7). Além disso, até o final do século XX, os mapas eram analisados segundo critérios europeus, marginalizando formas não tradicionais de representação e, sobretudo, derivando uma posição científica ortodoxa que colocava no centro da análise as semelhanças entre os mapas, descartando as diferenças (Harley, 1991).

Ao analisar os mapas dos séculos XVIII e XIX, o Exército Brasileiro teve como objetivo verificar a possibilidade de um consenso na demarcação de divisa entre Piauí e Ceará. Se considerarmos os objetivos históricos utilizados para a confecção de elementos cartográficos até o século XIX, assim como seus métodos, torna-se evidente o motivo de o relatório apontar uma indefinição: “A serra da Ibiapaba foi representada de forma iconográfica, à mão livre, o que, por consequência, nos permite concluir que sua delimitação possui maior caráter informacional do que métrico” (Brasil, 2024, p.45). Isso porque a serra da Ibiapaba aparece ora com divisa traçada no centro, ora pelas bordas; no mais das vezes nem sequer aparece delimitação alguma. Essas variações foram observadas em mapas encomendados tanto no Piauí quanto no Ceará.

Ora, permanece aqui uma visão insistente na noção de mapa que ignora os elementos constitutivos das representações cartográficas em seus contextos, buscando na sobreposição de mapas históricos qualquer espécie de ponto comum que permita traçar a linha divisória entre os estados. São posturas que a cartografia crítica já demonstrou serem errôneas; os pressupostos de construção dos mapas históricos tornam impossível qualquer tentativa de comparação demandada apenas pelas similitudes, e, em alguns casos, é justamente nas diferenças sobrepostas que residem os dados fundamentais para a análise. Propomos, portanto, uma análise de alguns mapas selecionados do arquivo digital da Biblioteca Nacional brasileira, os quais contrastaremos com documentação histórica e com a perspectiva de uma cartografia crítica moderna, a fim de traçarmos parâmetros para o uso da cartografia histórica na disputa entre Piauí e Ceará.

Dividimos esse exercício em três seções. Na primeira, buscamos explicitar as relações de poder inseridas nas representações cartográficas e os contextos que permeiam as diferenças e semelhanças ao longo dos séculos XVIII e XIX, o que se faz perceber desde o título retirado de uma das fontes. Depois, recuamos o recorte temporal para entendermos as motivações que levaram às disputas fronteiriças, dentro de um quadro sociopolítico obediente às diretrizes da Coroa portuguesa: conhecer e ocupar. Por último, utilizamos uma referência a Roger Chartier (2024) como provocação para fixarmos a complexidade contida na ambiguidade própria dos mapas. Apesar dos recuos temporais, a maioria dos mapas consultados no acervo da Biblioteca Nacional brasileira é do século XIX; a preponderância do Piauí no material aqui consultado justifica-se pelo fato de ser autor da ação judicial.

## “Muito útil ao interesse dessa província”

**Figura 1:**
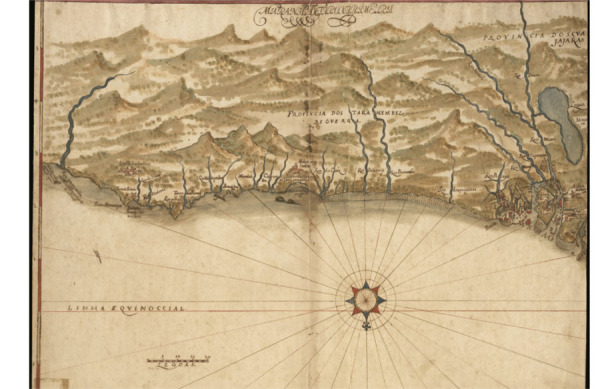
: Representação cartográfica do Estado do Maranhão e Grão-Pará, nessa época distinto do Estado do Brasil (Albernaz I, 1629)

Na transição da cartografia tradicional para a cartografia crítica podemos destacar a grande contribuição de Harley por inserir certo relativismo contextual na nova cartografia histórica da década de 1980, contestando as visões positivistas que entendiam os mapas como elementos estáticos. É bem certo que ainda hoje enfrentamos dificuldades em alcançar a profundidade dos mapas, mas podemos ao menos pressupor que não são, em si mesmos, nem verdadeiros, nem falsos. Sendo, ao mesmo tempo, linguagem, significado e produto social, misturam abstração e “imperativos territoriais de um sistema político”, com o adicional de um caráter vigilante aos casos de determinação de fronteiras, como o nosso (Harley, 2009, p.3). Para destacar o que queremos dizer, comecemos com um mapa que não está presente no relatório do Exército, datado de 1629, período da União Ibérica, quando o território que viria a ser o Piauí, apesar de já apresentar ocupação, ainda não tinha estrutura administrativa própria. Trata-se do *Pequeno atlas do Maranhão e Grão-Pará* (Figura 1), desenhado pelo cartógrafo português João Teixeira Albernaz I (1602-1662).

Albernaz pertencia a uma família de cosmógrafos, sendo hoje conhecido pela alcunha de “o velho” para se diferenciar de um neto homônimo que teve a mesma profissão. Em 1602, foi nomeado cosmógrafo do Armazém da Guiné e Índia, responsável por fazer e supervisionar a produção de cartas marítimas. Sua prolífica produção resultou em 19 atlas, totalizando mais de duas mil cartas com foco nas explorações marítimas e terrestres de Portugal, especialmente do Brasil. O mapa aqui analisado está orientado para o sul e chama a atenção pela descrição detalhada da costa entre o Ceará e o Pará, com o desenho dos principais rios que percorriam a região e de algumas rotas de navegação. No entanto, o interior não ostenta praticamente detalhes, exceto pelos nomes das etnias indígenas que seriam ali encontradas, além do caminho por terra que ligava o Maranhão ao Pará.

Note-se que há uma diferenciação na toponímia: há uma identificação para a “província dos Taramembez (Tremembés) de guerra” e outra para “aldeia dos índios”. No espaço entre o forte do Ceará e a ilha de São Luís há apenas um espaço costeiro com desenho pontilhado que pode significar obstáculos à navegação. A serra da Ibiapaba não tem representação regular, apresentando apenas uma cadeia de montanhas paralelas ao Atlântico. Essas observações revelam dois aspectos sobre o lugar: primeiro, o de ser território “desconhecido” no seu interior, que viria a ser reclamado posteriormente; e segundo, a “selvajaria” dos índios^1^ locais que justificaria os pedidos de posse de terra subsequentes. Em 1701, ainda ocorriam contendas nos sertões envolvendo índios e criadores de gado que haviam “descoberto [estas terras] com grande perigo de vida por estar toda povoada de gentio” (Consulta…, 26 fev. 1701).

**Figura 2:**
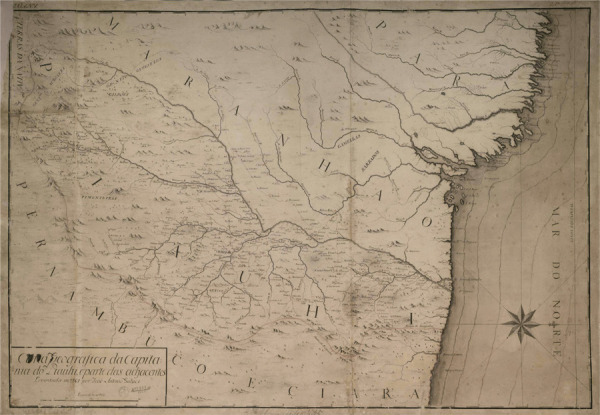
: Representação cartográfica da capitania do Piauí, demonstrando um importante espaço de ligação entre o estado do Maranhão e o Estado do Brasil (Galluzzi, 1761)

Os gentios seriam preocupação constante durante todo o processo de ocupação do território, e, geralmente, os pedidos de sesmaria se justificavam na expulsão desses gentios para rendimento de dízimo (Requerimento…, 14 mar. 1726). Esse movimento pôde ser observado no processo de ocupação de todo o interior do Piauí, sendo que, em um mapa de 1761 (Figura 2), já se apresentava uma capitania detalhadamente descrita. Trata-se da representação publicada por João Antônio Gallucio, uma reedição de outro mapa publicado no ano anterior por Henrique Antônio Gallucio (ou Galluzzi). Este último era italiano e engenheiro militar, fez parte da comissão responsável pelas demarcações geográficas resultantes do Tratado de Madrid, mas permaneceu no Brasil com outros do grupo, respondendo às políticas pombalinas e executando trabalhos em todo o Grão-Pará e Maranhão, a quem o Piauí, embora já criada a capitania, permanecia dependente administrativamente.

Na *Carta geográfica da capitania do Piauí e parte das adjacentes*, Galluzzi (1761) desenhou as fronteiras políticas do Piauí seguindo os cursos do rio Parnaíba, separando-a do Maranhão, e da serra da Ibiapaba, que ficava no lado do Ceará e de Pernambuco. O mapa está orientado pelo oeste e também faz referência aos gentios (Acroazes e Gueguezes), mas estes já estão representados no extremo sul do território, o que indica uma interiorização da ocupação já bem avançada, com detalhamento dos rios e seus afluentes. No que se refere às capitanias adjacentes, os litorais do Maranhão e do Pará têm as localidades detalhadas, o que pode revelar uma conexão de interdependência, já que o litoral do Piauí era utilizado principalmente para navegação de cabotagem, e informações de relevo estão presentes com maior precisão.

Embora a capitania do Piauí tenha sido desanexada do Maranhão em 1718, o ato só teve execução em 1758, e a capitania só se fez de fato independente em 1811. O *Mapa das cidades, vilas, lugares e freguesias das capitanias do Maranhão e Piauí* revela bem essa dependência administrativa. Elaborado a pedido do então governador do Piauí, José Telles da Silva, o mapa é, na verdade, uma espécie de censo, publicado em 1787 (Mapa…, 1787), interessado particularmente no aumento da população, mas com outras informações relevantes, como a distância de diversas localidades até as respectivas capitais, o número de nascimentos e de mortes etc. Nota-se a permanência da preocupação em indicar lugares de índios, dessa vez quantificando-os: havia, nas duas capitanias, 57.556 escravos de ambos os sexos, 1.145 mulatos e pretos forros, 9.804 índios e 30.238 brancos.

Apenas as diferenças entre os dois mapas que reproduzimos aqui, entre a primeira metade do século XVII e a segunda metade do século XVIII, são suficientes para pôr em questão a própria intencionalidade dos governos que mandavam fabricar seus mapas, mas essa relação também não é óbvia como parece. Por exemplo, no *Mapa geográfico da capitania do Ceará*, elaborado por Mariano Gregório do Amaral, em 1800, o objetivo era cartografar o Ceará, e a linha de divisa está totalmente no ocidente em relação à Ibiapaba. Já na *Carta geográfica do Piauí*, feita por José Pedro Cezar de Menezes, em 1809 – embora encomendada por Carlos Cézar Burlamaqui, então governador do Piauí, para correções do mapa de 1760 –, a linha também está no sopé ocidental. A *Geographische Karte der Provinz von Ciará*, elaborada por Jos Schwarzmann e Le Chev de Martius, em 1831, não delimita a serra da Ibiapaba e a representa de maneira difusa, muito embora detalhe vias para fluxo de pessoas nas localidades sobre a Ibiapaba em direção às duas províncias.

A *Carta topográfica e administrativa da província do Piauí*, feita pelo visconde J. de Villiers de L’Isle Adam, em 1850, apesar de representar a província do Piauí, nem sequer demarca o leito do rio Poty, importante referência nos limites com o Ceará, e ignora aspectos presentes nos mapas anteriores, tais como os rios que corriam da serra da Ibiapaba para o Piauí. A *Carta corográfica da província do Ceará*, organizada pelo engenheiro Antônio Gonçalves da Justa Araújo, em 1881, é a primeira a sinalizar mudança na divisa das províncias, entendida pelo Exército como a tentativa de conformar o mapa ao decreto imperial n.3.012, de 1880, por conta do ano de publicação – o que pode não se confirmar, visto que Gallucio, por exemplo, reclamou do curto período de um ano para desenhar um mapa, a não ser que reeditasse mapas anteriores apenas inserindo a nova informação.

O problema que persiste é: mesmo em mapas do século XX, com novas tecnologias disponíveis para uso da cartografia, produzidos na mesma época e pelo mesmo órgão governamental, sobressaem diferenças de divisas, rios, povoados e formatos, como aponta o relatório do Exército. Não se pode perder de vista que essas cartas atendiam a objetivos bastante específicos de conhecimento e posse em seus contextos, e indicavam pontos precisos que, mesmo que não tivessem precisão métrica nas cartas, existiam na paisagem e por isso não deixavam de demonstrar caráter métrico também. Podiam apresentar, inclusive, distorções propositais para atingir seus fins. Isso talvez explique a observação do Exército de que não há áreas de litígios representadas nos mapas históricos analisados, visto que não era esse o escopo deles.

Ora, se a análise do Exército Brasileiro concluiu que a cartografia histórica não tem finalidade métrica, então qual o motivo da sua utilização? Ao largo de demonstrar as intenções particulares de cada um, o que a análise possibilitou ao Exército foi uma inferência que considerou as coincidências encontradas na maioria dos mapas. Assim, dos 15 mapas históricos analisados pelo Exército entre 1760 e 1881, nove representam a linha de divisa das capitanias/províncias do Piauí e do Ceará a oeste da vila da Amarração; 11 representam a serra da Ibiapaba afastada do litoral; 11 representam a nascente do rio Timonha a leste da serra da Ibiapaba; dez apontam que a linha de divisa não toca o leito do rio Poty. Pontos de localização em comum permitiram ao Exército concluir que a maioria dos mapas representa a divisa predominantemente na encosta oeste da serra da Ibiapaba, não pelo divisor de águas em seu centro, como requerido pelo Piauí na disputa atual.

No entanto, as inferências não cessam o jogo de imprecisões que persiste desde o período colonial, do qual nasce a falta de conhecimento dos limites do estado do Maranhão (ao qual pertencia o Piauí) com o Ceará, e alguns detalhes de linguística dão espaço para recursos. Na análise do Exército sobre o decreto imperial de 1880, reconhece-se a imprecisão da divisão que concedia ao Ceará as porções orientais da serra, pois o trecho afirmava que a divisão procedia “nesta parte” específica onde se localizava o Boqueirão, dando a entender que não em toda a fronteira. Tudo isso nos leva a pensar como Magnus Roberto Pereira (citado em Kury et al., 2012) acerca da construção de conhecimento científico no século XVIII, de onde partem nossos questionamentos: aqui, os projetos iluministas do período colonial, como a utilização dos recursos naturais, confluem em problemas que, ao mesmo tempo antigos e atuais, permanecem ao longo do tempo. Nem sempre era possível levar até o fim os processos de produção de conhecimento, o que gerou certos “vazios” e diversas disputas (Pereira, citado em Kury et al., 2012, p.157). Um recuo temporal pode ajudar-nos a contemplar com maior clareza como ambiente e política se misturam nas representações cartográficas, carregando-as de intencionalidades.

## “Ao bem comum dos seus vassalos, e ao sólido aumento do Estado”

O recuo histórico nesse tema é inevitável, porque a preocupação com a demarcação do território entre Piauí e Ceará pode ser encontrada em documentos do século XVIII. Um dos relatos foi escrito em 1745 por Mathias Botelho, ouvidor-geral da então capitania do Piauí, e descreve os problemas de jurisdição na divisa do rio Poty, na serra dos Cocos e na ribeira dos Crateús, área importante para a demarcação da serra da Ibiapaba nos mapas do século XIX (Carta…, 24 ago. 1745). Dirigida ao rei D. João V, a carta do ouvidor denuncia a postura de um capitão-mor do Ceará que entrou nos distritos “publicando que aquela serra dos Cocos e seus anexos não era pertencente a esta capitania [do Piauí], nem ao Governo do Maranhão; fazendo prisões e expedindo ordens”, respaldando “habitadores [que] deram na ideia de quererem equivocar as jurisdições para fazerem mais desordenados os seus malefícios”. Como resultado disso, os ânimos alterados dos moradores da região fizeram com que não obedecessem ao Ceará, “cuja distância faz menos vigorosos os preceitos”, e questionassem a jurisdição do Piauí, criando um limbo espacial onde a arbitrariedade isentava os moradores das obrigações, com prejuízos à Real Fazenda, de acordo com as queixas dos contratadores de dízimos (Carta…, 24 ago. 1745).

Em 1761, o governador do Piauí, João Pereira Caldas, escreveu ao secretário de Marinha e Ultramar, Francisco Xavier de Mendonça Furtado, e recordou que ouviu o próprio secretário usar a serra da Ibiapaba como divisor entre o Piauí e Pernambuco, mas como não encontrou documentos que comprovassem essa demarcação, pediu providências contra a invasão das Justiças de Pernambuco e do Ceará na jurisdição que o Piauí exercia “em terras que inteiramente se acham situadas nas vertentes que faz a dita serra para este governo”. Nesse ofício, podemos encontrar explícita, além da inexatidão na divisa, a motivação do pedido: “seria muito útil ao interesse” do Piauí se fosse sujeita ao seu governo “aquela grande povoação de índios que há no alto da referida serra”, pois oferecia a “conveniência” de ser abastada de trabalhadores, uma vez que o governador reclamava dos “poucos índios” que se conservavam na capitania, e poderiam ser “acudidos” com “mais prontas providências” dadas as distâncias. Mais, Pernambuco não sentiria falta com essa separação, por causa das “muitas povoações da mesma qualidade de gente” com que contava (Ofício…, 16 set. 1761).

Nove anos depois, em alvará de 1770, o rei D. José anexou a vila Viçosa Real, povoação indígena então pertencente a Pernambuco, e mais duas povoações indígenas do Maranhão ao Piauí. O argumento lembra o mesmo que foi utilizado por Caldas alguns anos antes – administração da Justiça, “de que depende a paz e a tranquilidade pública dos povos”. Considerando a distância de trezentas léguas entre Vila Viçosa e a capital de Pernambuco, assim como a distância entre a freguesia de São Bento dos Pastos Bons (ou das Balsas, onde se localizavam as duas povoações dos índios Amanajos) e a capital do Maranhão, foi preferível ao rei “a grande facilidade com que os moradores da dita Vila podem recorrer à Cidade de Oeiras, capital do Piauí”. Resolvia-se assim o problema dos “vassalos vexados com as violências” proporcionadas pelas grandes distâncias (Alvará…, 28 jul. 1770).

Não era a primeira vez que o tema vinha à tona. Em 1720, uma consulta do Conselho Ultramarino ao rei sobre as petições do procurador das Missões do Brasil, padre João Guedes, indicava os danos que a aldeia da Ibiapaba sofreria se retirada da jurisdição do Ceará. O Conselho recomendava suspender a anexação da área ao Piauí, que se deu a partir da representação do mestre de campo Bernardo de Carvalho de Aguiar, a quem foi encarregada a guerra contra os gentios bravos do Piauí. Os índios se mostravam “sumamente desconsolados” com essa mudança, porque já haviam sofrido, alegavam, dois anos e meio debaixo do jugo do Piauí, cuja tirania os obrigou a fugir e se meterem nos matos em que estiveram por trinta anos. Argumentava ainda que os índios, sozinhos e sem ajuda de brancos, “destruíram” os Tapuias^2^ que ocupavam densamente o Piauí, pelo que pediam armas e autonomia (inclusive com a permissão de conceder paz e alianças), pois só eles podiam penetrar os matos e sertões e defender as fronteiras (Consulta…, 16 out. 1720).

Nada era inédito. Pouco antes de 1720, os índios da serra da Ibiapaba escreveram ao rei solicitando o alargamento de suas terras, pois as que possuíam por demarcação eram penedias e “quebradas inúteis”. Cada vez mais Tapuias se juntavam à aldeia, pelo que somavam mais de quatrocentas almas, e as poucas terras em que eram capazes de plantar já estavam cansadas, levando à fome, que só se resolvia pelo pouco gado criado pelos missionários. A solução alcançava principalmente os mais necessitados, mais de cem viúvas e muitos meninos órfãos “cujos pais morreram nas guerras, ou de doenças em climas estranhos em serviço”. Pediam as terras que ficavam da ladeira do Uruoca até o lugar Itapiúna. Além disso, reclamavam da ausência de homens, ocupados o ano inteiro em serviços dos missionários. Eram índios originários da Bahia, de onde foram para a Ibiapaba com outros povos que se estabeleceram na serra do Araripe, depois de se separarem na passagem do rio São Francisco – poderiam ser mais de quatro mil almas, e os índios da Ibiapaba usavam isso a seu favor, solicitando permissão para encontrá-los e aumentarem o número de vassalos do rei (Requerimento…, 12 out. 1720).

Os índios da Ibiapaba já estavam acostumados ao serviço e estabeleciam contatos em busca de alianças. Em 1728, próximo ao mês de setembro, a vila da Mocha (depois Oeiras, capital do Piauí) foi invadida por Timbiras (índios de guerra), e o governador dos índios da Ibiapaba, que estava na vila quando do ocorrido, socorreu-a com cem soldados (Carta…, 25 set. 1728). Isso não excluía a capacidade de agência dos próprios índios ou dos missionários: pouco antes, em 1725, o governador de Pernambuco respondeu ao pedido do rei para enviar índios da Ibiapaba para o Maranhão, afirmando que não podia atendê-lo, porque o Piauí estava habitado intensamente de gentios bárbaros, e, se estes soubessem que os índios estavam ausentes, podiam atacar. Além disso, precisavam cuidar de suas lavouras, pois experimentavam grande fome (Carta…, 10 jul. 1725).

Eram ativos também quanto às questões de terra. Em 1753, iniciaram uma luta contra os moradores do entorno da Ibiapaba, atacando casas a mais de vinte léguas da missão, de proprietários que residiam ali há trinta anos, criando gado, lavoura e pagando dízimo real (Carta…, 12 nov. 1753). Três anos depois, o rei solicitou informações sobre a contenda, e o governador de Pernambuco informou que a circunvizinhança estava em perfeita paz e que, por isso, não deveria mexer nesse assunto (Carta…, 15 maio 1756). É provável que os índios tenham feito alianças e acordos com os moradores da região, pois aumentaram em número, e a demanda por terra deveria também ter aumentado: em 1708, eram quatrocentos casais de Tabajaras e Acoansus, com duas mil almas, fora Tapuias; em 1756, eram mil casais de Tabajaras, Tapuias, Agoanacés, Guacongoaçus e outros, com 6.106 almas, sem contar os que há anos estavam fora (Certidão…, 13 fev. 1756; Carta…, 13 fev. 1708).

O que podemos inferir daqui é que os habitantes da região da Ibiapaba estavam inseridos na lógica de possessão portuguesa e reivindicavam suas necessidades, por isso fugiam à alcunha de gentio bravo. Mas esses esforços não podem ser compreendidos sem os missionários que primeiro estudaram esses locais e seus caminhos. Valquíria Ferreira da Silva (2021) demonstra como as práticas dos jesuítas estavam profundamente emaranhadas com o conhecimento do mundo natural. Não ironicamente, num mundo cada vez mais matematizado nas representações, todo matemático entre os séculos XVII e XVIII estava ligado aos jesuítas de alguma maneira, e, por cultivarem o conhecimento de astronomia e matemática que dá suporte à cartografia, os jesuítas que vieram ao Brasil foram conhecidos como padres matemáticos. Furtado (2020) ressalta a proeminência desses missionários na circulação de conhecimentos geográficos de regiões ignoradas pelos europeus. A importância dos jesuítas, nesse caso, cresceu à medida que ocupavam uma área que interligava o Estado do Brasil ao estado do Maranhão, em regiões do Ceará onde “os portugueses haviam realizado ‘apenas investidas sem permanência. O sertão continuou fechado’” por muito tempo (Silva, 2021, p.84).

Temos, portanto, configurações políticas bastante complexas na serra da Ibiapaba, com parte de seu território sob jurisdição do Estado do Brasil e outra sob o governo do Maranhão e Grão-Pará. Quando os jesuítas foram expulsos da região, em 1661, deixaram as incursões sob demanda do Estado do Brasil. Antes disso, os empreendimentos missionários receberam incentivo, porque essa zona de transição era tida como instável, mas peça fundamental para a construção de um caminho que unisse os dois estados, elevada a uma “questão máxima” nas últimas décadas do século XVII (Silva, 2021, p.193). As populações indígenas ocupavam lugar central, já que até então a comunicação por terra era impraticável por conta das dificuldades naturais e, sobretudo, das hostilidades. Cumprindo um duplo papel, os missionários catequizavam os índios, inserindo-os no universo luso-brasileiro, e utilizavam-se deles como mão de obra.

Eram esses jesuítas que apresentavam às autoridades um território conquistado, conhecido e civilizado. Essa postura estava diretamente subordinada aos interesses da Coroa, que, após a expulsão dos missionários jesuías, editou, em 1757, o *Diretório que se deve observar nas povoações dos índios do Pará e Maranhão*, não por acaso tendo como conselheiro Francisco Xavier de Mendonça Furtado, ministro plenipotenciário nas conferências de demarcação dos limites setentrionais do Estado do Brasil. Buscava inserir os índios nos meios da “civilidade, da cultura e do comércio”, concorrendo para o “sólido estabelecimento do Estado” por meio, primeiramente, do cultivo das terras.

Quase ao mesmo tempo, o engenheiro Henrique Antônio Gallucio (ou Galluzzi) foi mandado para o Pará, na frota de 1753 a serviço da Coroa portuguesa, pelo período de cinco anos como ajudante de Infantaria, mas com exercício de engenheiro e soldo dobrado. Nessa ocasião fez um mapa topográfico da região e abriu um caminho por terra para que os moradores da vila de Bragança se comunicassem por linha direta com a cidade do Pará (Belém), além de corrigir marcações do rio Negro com observações astronômicas de latitude e longitude. Cumpridos os cinco anos, Gallucio passou a trabalhar no pedido régio de um mapa da capitania do Piauí, executando, primeiramente, a medição da costa marítima do Pará até o Maranhão. Alegou ter cumprido a tarefa no “limitado prazo” de um ano, sem auxílio de outra pessoa, exercendo não menos a astronomia que a engenharia e, diante de todas as dificuldades expostas, solicitava progressão ao posto de sargento-mor engenheiro com respectivo aumento de soldo (Carta…, 23 nov. 1760).

Gallucio ressaltou que nas suas diligências, desde a configuração geométrica da costa marítima até o Parnaguá (extremo sul do Piauí), superou chuvas, a “infestação do gentio”, “caminhos não praticados”, além de uma “grave doença adquirida na derradeira viagem”. Mas executou as medições repetidamente, para evitar equívocos, e o fez com “extraordinário cuidado”, mesmo quando “cercado das maiores dificuldades e metido nos mais evidentes perigos”, pois contava com a esperança de ter seu trabalho premiado (Carta…, 23 nov. 1760). Estamos diante dos primeiros esforços para demarcar a região de forma mais precisa, dentro de um território com fronteiras políticas internas ainda instáveis no estado do Grão-Pará e Maranhão (que compreendia as capitanias de São José do Rio Negro, Grão-Pará, Maranhão e Piauí).

Em sentido mais abrangente, trata-se do processo de interiorização econômica, motivado pela mineração e pecuária, que criou também vias de comunicação, mesmo que em precários corredores que interessavam à Coroa, em busca de salitre e outros recursos naturais, por exemplo, e assim foram cartografados, estudados e fiscalizados (citado em Kury et al., 2012, p.16). Não era estranho encontrar núcleos urbanos sertão adentro, e Piauí, Maranhão, Pernambuco, Ceará e Goiás estavam diretamente ligados por estradas e pelos principais rios, o Parnaíba e o Tocantins, sendo possível aproximar-se inclusive do São Francisco e, consequentemente, da Bahia, em rotas comerciais que não deixavam a região em completo isolamento (Silva, 2016, p.107). Essa dinâmica fez com que “veredas de vaqueiros” se transformassem em “trilhas articuladoras de todos os tipos de negócios”. Diante de tamanha conexão, não é de se estranhar que as fronteiras políticas acabassem se confundindo, e, se no âmbito social as relações de poder perpassam as definições de domínio, na espacialidade a demarcação de fronteiras faz-se essencial (citado em Kury et al., 2012).

## “A agradável verdade do inverossímil”

Em interessante artigo, Iris Kantor (2022) recordou a dificuldade de gerência nas expansões internas do Brasil desde a intensificação das suas representações com o fim do tráfico transatlântico até o pós-emancipação, numa perspectiva de longa duração. Dessa maneira podemos ver como a unidade geopolítica da América Portuguesa dependeu, em grande medida, da acumulação de experiências nas próprias expedições demarcadoras, como vimos no caso dos missionários jesuítas e do engenheiro Gallucio, levando a soberania portuguesa à realidade local. Descrever o espaço físico e suas riquezas era de fato uma prioridade na inserção desses espaços na administração portuguesa e foi tarefa prioritária para toda uma geração de cientistas e agentes.

A produção de mapas, ao longo dos séculos XVIII e XIX, seguiu incorporando diversas tecnologias, mas continuou – e continua – dotada de intenções que não são simples de destrinchar e, sobretudo, não devem indicar um progresso linear na cartografia. No século XIX, encontramos uma cartografia aprimorada, porém, tributária do século anterior. Por exemplo, o *Mapa geográfico da capitania do Piauí, e parte das do Maranhão, e do Grão-Pará*, (Mapa…, [1816?]), de autoria desconhecida e oferecido ao governador do Piauí Balthazar de Souza Botelho de Vasconcelos, provavelmente em 1816, apresenta inúmeras semelhanças à carta de Gallucio, com o acréscimo de caminhos entre as povoações e a indicação de fazendas de gado. É provável que seu autor tenha utilizado essa carta como base, atualizando-a com novas informações disponíveis. Trata-se já de uma capitania administrativamente independente, ainda que profundamente conectada com o Maranhão.

Por outro lado, o *Guia dos caminhantes* desenhado e escrito por Anastácio de Sant’Anna, com alcunha de Pardo Velho pintor, em 1817, foi justificado pela correção de erros contidos em atlas anteriores, acrescentando que as suas ilustrações propiciariam o conhecimento do mundo àqueles que não têm meios de viajar, e ofereceu seu resultado aos comerciantes, fazendeiros e leitores em geral, com notáveis imprecisões e deformações. Quatro mapas do conjunto fazem referência ao Piauí, mas todos representam a capitania de forma distorcida. Na sétima carta, o Piauí aparece como um corredor que se estende entre o Maranhão e o Ceará, separando-os; enquanto na carta oitava, o rio Parnaíba é representado mais próximo do Ceará que do Maranhão. A exemplo dos mapas chineses destacados por Harley (2009), essa carta do Pardo Velho não era puramente um instrumento de medição, mas repleta de literatura, imagens, pinturas e conhecimentos gerais, fugindo ao caráter cristalizado que uma cartografia histórica tradicional poderia lhe relegar, sendo exemplos mais claros as cartas primeira e oitava.

Talvez o mapa mais completo do período em questão seja a *Geographische Karte der Provinz von São Iozé do Piauhý*, do alemão Joseph Schwarzmann, impresso em Munique, em 1828. Existem várias outras cartas do autor no acervo da Biblioteca Nacional brasileira, mas dele não obtivemos mais informações além de estar relacionado aos nomes dos viajantes naturalistas Spix e Martius. Há referência aos manuscritos de Jozé Pedro Cezar de Menezes e Mathias Jozé da Silva Pereira, tendo o primeiro bastante experiência em cartografar o Piauí. Permanecem indicações sobre os índios Guegues e Pimenteiras, além de poucos espaços em branco no sul da província, na direção de Goiás e de Pernambuco – após esse período, as populações indígenas tornam-se rarefeitas nas representações, a exemplo da *Carta topográfica e administrativa da província do Piauí*, “erigida sobre os documentos mais modernos pelo visconde J. de Villiers de L’Isle Adam”, na qual não se observa nenhuma indicação de aldeamento. Além disso, a *Geographische Karte* é pouco precisa no que diz respeito aos limites fronteiriços com a província do Ceará.

No pós-independência encontramos explícito o tema da demarcação de fronteiras na *Carta corográfica do território que o Ceará deve restituir ao Piauí pelo lado da freguesia da Parnaíba*, levantado por David Moreira Caldas, de 1868, provavelmente encomendado pelas autoridades do Piauí. Já no século XX, prevalece o processo de inserção do interesse militar na cartografia, em seu conteúdo e na sua produção, com destaque para o relevo, estradas de rodagem, estradas de ferro e pontos de pouso, a exemplo do *Mapa dos Estados do Maranhão, Piauí, Ceará, Rio Grande do Norte, Paraíba, Pernambuco e Alagoas* (Brasil, 1942). Esse caminho que a cartografia percorre no caso em questão, ao reconhecer os elementos sociopolíticos presentes em cada um dos mapas aqui elencados, demonstra como essas cartas se tornaram mais esquemáticas, sem deixar de lado o caráter simbólico do poder da representação e de seus interesses.

No caso do sertão do Piauí, tais interesses estavam intrinsecamente ligados ao comércio, que exigia abrir caminhos, estabelecer vilas e conectar as pessoas e os espaços. Percebemos como a rede de interdependência com as províncias vizinhas influenciou as representações cartográficas. Da mesma forma, sobressaltam os povos indígenas, extremamente úteis aos interesses da administração pública, como apontado na carta do governador do Piauí em 1761, mas gradualmente excluídos da cartografia. Talvez a reclamação do governo sobre a falta de índios no território, vista anteriormente, reflita a inserção desses povos nos moldes civilizatórios, já que aqueles que resistiam eram descritos como gentios bravos, e não como índios (Carta…, 10 jul. 1725). Podemos estar diante do silenciamento das resistências indígenas no Piauí, dentro do processo de entendimento do espaço que exerceu poder sobre essas populações.

Tornando ao plano mais amplo, se tomarmos os mapas não como verdadeiros ou falsos, mas como estruturação do mundo dos homens, segundo a qual se projetam culturas na lógica dos Estados modernos (Borges, 2002), notamos que o espaço do Piauí deixou de ser simplesmente um território para se configurar como uma província independente, com suas próprias reivindicações (Sousa, 2022). Se não raro os mapas precedem os territórios, a exemplo dos mapas do Brasil que alteravam os limites reais do Tratado de Tordesilhas (Furtado, 2012), a questão que se segue é a seguinte: conhecendo as razões que levaram os homens a intervirem e representarem o espaço do Piauí e requererem expansões internas devidamente justificadas, continuam essas justificativas válidas nas questões da atualidade? Os mapas podem adquirir significados diversos, sendo construções sociais do mundo de quem os lê, da forma como se lê, recheados de subjetividade e de uma linguagem gráfica que se deve decodificar (Harley, 2005). O que devemos questionar é se toda essa complexidade está sendo levada em conta na utilização da cartografia histórica para a resolução da disputa territorial que se iniciou ainda no século XVIII, e como esse litígio se transformou ao longo da história.

A título de conclusão parcial em seu relatório, o Exército localizou a indefinição de limites na data do decreto imperial de 1880, o que, conforme vimos, não é totalmente correto, já que reivindicações anteriores datam de pelo menos 1745. Assim como não é completamente adequado atribuir à cartografia histórica o caráter simplesmente informacional, já que essas cartas foram utilizadas pelos governos ao longo do tempo para implementação das mais diversas políticas. Mas o levantamento de campo também traz dados atuais e relevantes, tais como a presença de prédios de ensino (43 do Ceará e vinte do Piauí), edificações de saúde (nove do Ceará e três do Piauí), depósitos de abastecimento de água (182 do Ceará e 87 do Piauí) etc. Em termos populacionais, o somatório da população dos municípios envolvidos é de 674.995 habitantes, segundo o Censo Demográfico de 2022.

O documento do Exército aponta cinco possibilidades de divisão, quais sejam: (1) considerar o divisor de águas da serra da Ibiapaba como marco divisório; (2) dividir as zonas de litígio em áreas equivalentes; (3) demarcar o limite na linha oeste da serra; (4) demarcar o limite na linha leste da serra; (5) compilar resultados, considerando como limite a divisão do Instituto Brasileiro de Geografia e Estatística. Na primeira opção, seriam atingidos 268.222 habitantes do Ceará e 876 habitantes do Piauí, e essa divisão contraria os mapas históricos e a ocupação territorial, segundo o Exército. Na segunda opção, o Ceará receberia uma quantidade maior de edificações que o Piauí, com uma população total de 28.886 habitantes diretamente atingidos. Na terceira opção, o Ceará seria o maior impactado, tendo em vista que é a parte que contém a maior área nas zonas de litígio, e todos os 2.820km^2^ seriam incorporados ao Piauí. Na quarta sugestão, o Piauí seria impactado negativamente, pois mesmo sendo o Ceará detentor da maior participação nas áreas litigantes, o Piauí tem maior ocupação em duas regiões específicas. Por último, a adoção da linha atualmente definida pelo IBGE não afetaria a população ou a distribuição de edificações nos dois estados, refletindo a ocupação humana das áreas em litígio e sendo necessária apenas a definição de uma divisa final considerando as áreas inconsistentes encontradas no trabalho de campo do Exército.

Daqui derivam duas observações a respeito da concretude das representações e da influência da cartografia na vida cotidiana. Se olhar para um mapa implica enunciados de materialidade do mundo (Jacob, 2016), a descoberta de limbos espaciais além das áreas em litígio entre Ceará e Piauí, importantíssimo resultado da análise do Exército Brasileiro, pode indicar a agência do ambiente diante da tentativa de controle do mundo natural típica do século XIX luso-brasileiro, além de evidenciar que mesmo as cartas geográficas elaboradas por satélites não deixam de ser uma construção social. E, neste sentido, é crível que realmente a nossa relação com o mundo permanecerá alienada enquanto não entendermos que cultura e natureza são “o mesmo conceito separado em suas partes que se encontram ligadas” (Latour, 2020, p.34).

Como afirma Harley (2009), os mapas têm a mesma capacidade dos relógios de influenciarem de maneira invisível a vida cotidiana, limitando-a de acordo com os traçados que adquiriram, ao longo do tempo, maior caráter científico. Sabemos há muito tempo que os mapas têm consciência de seus desvios e distorções; resta-nos reafirmar a necessidade de ler os discursos implícitos nessas cartas, buscando as implicações políticas que daí derivam. Sobretudo, se Harley (2009, p.20-23) tem razão ao afirmar que os mapas, diferentemente da literatura ou outros tipos de arte, não comportam expressões populares ou atos subversivos, temos aqui a oportunidade de impedir que se despersonalize o território representado. Como vimos, nunca se tratou de um espaço socialmente vazio, humanos (e animais) ocuparam a paisagem, e não podemos continuar a desconectar as decisões de poder dos contatos interpessoais.

Aparentemente, o choque da descoberta de novas terras foi substituído pela crise daqueles que ainda buscam “ilhas desconhecidas”, demonstram as tensões geopolíticas que não cessam de eclodir. É urgente aceitar que o território que ocupamos ultrapassa as representações bidimensionais de qualquer mapa, sendo “aquilo do que dependemos para subsistir, aquilo que somos capazes de explicitar ou visualizar, aquilo que estamos prontos para defender” (Latour, 2020, p.410). Superando essas limitações, seremos capazes de encontrar saídas para a geopolítica das regiões em litígio – não apenas Piauí e Ceará – com uma visão realista, coerente e atual de pertencimento, deixando de lado uma cartografia preocupada em “fazer guerra” e aprendendo com “toda essa complexidade que os geólogos souberam administrar tão bem para determinar a longa história de solos e rochas, da qual a desafortunada geopolítica permanece desprovida” (Latour, 2020, p.428-429) – a materialidade do mundo.

Em seus escritos sobre o uso de mapas em obras de ficção, Roger Chartier (2024) convida a um interessante reconhecimento das motivações cartográficas que não encontram referência na realidade, tornam extremamente tênue a divisão entre objetividade e subjetividade e realçam o jogo “entre dispositivos de credibilidade e desmentidos de sua veracidade”. Embora geografia e fábula sejam aqui relacionadas ao limite, o autor busca na etimologia razões para explicar essa relação. Em francês, a palavra “mapa” não mantém uma distância considerável entre o figurado e o concreto: “Uma expressão muito comum em nossa língua diz ‘conhecemos bem o mapa deste lugar’, o que significa que sabemos como nos portar em determinado lugar ou situação”. Também se diz que um homem conhece bem o mapa para significar que ele está informado das “intrigas, interesses e maneiras do mundo” (Chartier, 2024).

O que essa ambiguidade reflete em nós é a negação de qualquer dicotomia. O mapa noticiado no UOL como “prova” que resolveria a questão, indicado no início deste artigo, não foi tratado no relatório do Exército, mas sua “descoberta” tampouco resolve a questão exatamente por sua incapacidade de precisar historicamente em qual parte da serra da Ibiapaba se dividem Piauí e Ceará: não era essa a sua função. Parece tratar-se de um mapa com autoria do cartógrafo inglês Aaron Arrowsmith, com poucos dados além do ano de 1840. Visto que caberia uma análise mais detalhada para saber se a publicação era uma atualização de mapas anteriores, com dados de outros cartógrafos da família Arrowsmimth, por exemplo, também não utilizamos esse mapa aqui. A crer na argumentação que endossa o mapa de 1840, continuaríamos a tratar os mapas como “espelho do mundo”, onde as ilhas desconhecidas se acabaram há muito tempo.

Diante desses dois usos de mapas históricos – um uso público, com pretensão de validação, amplamente noticiado; e outro uso técnico, de difícil acesso, em forma de relatório – temos também duas visões distintas que acabaram por se sobrepor na questão específica do litígio entre Piauí e Ceará: a verdade absoluta pretensamente contida no mapa e a descrença na precisão da cartografia histórica. O que a análise mostra em sua essência é o apagamento das comunidades indígenas enquanto os caminhos vão sendo abertos em direção ao interior e a consequente necessidade do Piauí em relação aos indígenas da Ibiapaba. Faltou ao relatório do Exército observar as relações de poder presentes na feitura de cada um desses mapas. Falta a nós – principalmente ao STF – incluir os povos originários da região para tomar qualquer decisão, de maneira subversiva e contrária aos rumos atuais do processo.

De maneira mais profunda, tratamos do trânsito de sujeitos que pretendiam o alargamento do conhecimento da natureza e que, para isso, foram capazes de estabelecer os mais complexos contatos com grupos locais (Kury et al., 2012, p.163), numa relação em que o mundo natural era agente transfigurado pelas representações. Portanto, a discussão não pode passar ao largo do ambiente e das pessoas que o ocupam. Se as disputas políticas deixaram as fronteiras suspensas no tempo ou na beira da incerteza, há que se recordar das populações diretamente atingidas para que não se corra o risco de cair no abstrato. A negociação do poder e as consequentes resistências justificam volver novos olhares, como o que tentamos aqui, para os povos originários das zonas conflitantes, que sempre estiveram ali, ocupando a dança desenhada nos mapas ao longo dos séculos.

## Data Availability

Não estão em repositório.

## NOTAS

1 A nomenclatura varia ao longo do texto porque escolhemos manter “índios” nos momentos em que se faz referência direta aos documentos. Em análises próprias, utilizamos “indígenas”, como convém atualmente.

2 De acordo com Márcio Roberto Alves dos Santos (2021, p. 51-52), o vocábulo “Tapuia” serviu desde o início da invasão portuguesa para designar os habitantes dos sertões, e significa “bárbaro”, ou “contrário”. Era uma denominação etnocêntrica que simplificava e reduzia todos os povos que não eram Tupi, quando estes negociavam com os portugueses. Tapuia, na documentação aqui trabalhada, acaba por significar o mesmo que “gentio bravo”. Ambos os termos são utilizados aqui quando fazem referência direta ao documento, com a ressalva de nomear os povos indígenas sem generalismo sempre que a documentação permitir.

## References

[B1] ALBERNAZ I (1629). João Teixeira. Pequeno atlas do Maranhão e Grão-Pará.

[B2] (1770). ALVARÁ do rei D. José anexando Vila Viçosa Real à capitania do Piauí.

[B3] BORGES Maria Eliza Linhares, Paiva Eduardo F., Anastasia Carla M.J. (2002). O trabalho mestiço: maneiras de pensar e formas de viver: séc. XVI a XIX.

[B4] BRASIL, Ministério da Defesa, Exército Brasileiro (2024). Relatório técnico referente à ação cível originária n.1.831.

[B5] BRASIL, Ministério da Guerra (1942). Mapa dos Estados do Maranhão, Piauí, Ceará, Rio Grande do Norte, Paraíba, Pernambuco e Alagoas.

[B6] CALDAS David Moreira (1868). Carta corográfica do território que o Ceará deve restituir ao Piauí pelo lado da Freguesia da Parnaíba.

[B7] (1760). CARTA de Henrique Antônio Gallucio ao secretário da Marinha e Ultramar sobre as configurações geométricas que efetuou no Pará, Maranhão e Piauí.

[B8] (1753). CARTA do capitão-mor do Ceará ao rei sobre as missões na Ibiapaba e as brigas entre índios e moradores por terra.

[B9] (1708). CARTA do desembargador Cristóvão Soares Reimão ao rei, sobre a vistoria feita à terra da aldeia dos tapuias "Acoansus" e índios Tabajaras na Serra da Ibiapaba.

[B10] (1725). CARTA do governador de Pernambuco ao rei sobre a ordem de mandar índios da Ibiapaba para o Maranhão. Códice 015, caixa 31, documento 2832.

[B11] (1728). CARTA do governador do Maranhão ao rei sobre a invasão dos índios Timbira à vila da Mocha. Códice 016, caixa 1, documento 40.

[B12] (1756). CARTA do governador do Pernambuco ao rei sobre as brigas entre índios da Ibiapaba e moradores, por terra.

[B13] (1745). CARTA do ouvidor-geral do Piauí ao rei D. João V sobre os problemas de jurisdição com o Ceará. Códice 016, caixa 4, Documento 238.

[B14] (1756). CERTIDÃO do número de índios da missão da Ibiapaba passada pelo padre João Brewer, visitador do Real Hospício do Ceará e missões a ele anexas.

[B15] CHARTIER Roger (2024). Mapas e ficções: séculos XVI a XVIII.

[B16] (1701). CONSULTA do Conselho Ultramarino ao rei D. Pedro II sobre povoadores e descobridores do sertão do Piauí. Códice 016, caixa 1, documento 4.

[B17] (1720). CONSULTA do Conselho Ultramarino ao rei sobre petições e representações que se referem aos danos que a aldeia da serra da Ibiapaba pode sofrer se retirar sua jurisdição do Ceará.

[B18] DE L'ISLE-ADAM J. de Villiers (1850). Carta topográfica e administrativa da província do Piauí.

[B19] FURTADO Júnia Ferreira, Pedley Mary, Edney Matthew (2020). History of cartography: cartography in the European enlightenment.

[B20] FURTADO Júnia Ferreira (2012). Oráculos da geografia iluminista: dom Luís da Cunha e Jean Baptiste Bourguignon D'Anville na construção da cartografia do Brasil.

[B21] GALLUZZI Henrique Antônio (1761). Carta geográfica da capitania do Piauí, e parte das adjacentes.

[B22] HARLEY John Brian (2009). Mapas, saber e poder. Confins.

[B23] HARLEY John Brian, Harley John Brian (2005). La nueva naturaleza de los mapas.

[B24] HARLEY John Brian (1991). A nova história da cartografia. O Correio da Unesco.

[B25] JACOB Christian (2016). Por uma história cultural da cartografia. Espaço e Cultura.

[B26] KANTOR Iris (2022). Os confins à vista nos mapas do Brasil. Ciência e Cultura.

[B27] KURY Lorelai Brilhante (2012). Sertões adentro: viagens nas caatingas, nos séculos XVI a XIX.

[B28] LATOUR Bruno (2020). Diante de Gaia: oito conferências sobre a natureza no antropoceno.

[B29] MADEIRO Carlos (2024). Mapa de 1840 achado em Londres vira prova no STF sobre litígio de CE e PI. UOL Notícias.

[B30] MALERBA Jurandir (2014). Acadêmicos na berlinda ou como cada um escreve a história? Uma reflexão sobre o embate entre historiadores acadêmicos e não acadêmicos no Brasil à luz dos debates sobre Public History. História da Historiografia.

[B31] MAPA das cidades, vilas, lugares e freguesias das capitanias do Maranhão e Piauí: com o número em geral dos habitantes das ditas capitanias e, em particular, de cada uma das referidas povoações, e da distância em que ficam da capital. [S.l.: s.n.], 1787. Mapa manuscrito, preto e branco, 62x48cm.

[B32] (1816). MAPA geográfico da capitania do Piauí, e parte das do Maranhão, e do Grão Pará.

[B33] (1761). OFÍCIO do governador do Piauí ao secretário da Marinha e Ultramar sobre a necessidade de demarcar a capitania.

[B34] (1726). REQUERIMENTO de João Gomes do Rêgo Barra ao rei D. João V, solicitando sesmaria junto ao rio Iguaraçu. Códice 009, caixa 15, documento 1499.

[B35] (1720). REQUERIMENTO dos índios da serra da Ibiapaba ao rei pedindo o alargamento de suas terras.

[B36] SANTOS Márcio Roberto Alves dos (2021). Rios e Fronteiras: conquista e ocupação do sertão baiano.

[B37] SCHWARZMANN Joseph (1828). Geographische Karte der Provinz von São Iozé do Piauhý.

[B38] SILVA Mairton Celestino da (2016). Um caminho para o Estado do Brasil: colonos, missionários, escravos e índios no tempo das conquistas do Estado do Maranhão e Piauí (1600-1800).

[B39] SILVA Valquíria Ferreira da (2021). Extraído do original: arte, ciência e técnica nos mapas da América portuguesa do padre Cocleo.

[B40] SOUSA Luis Carlos Albano Duarte (2022). Mestiços e corcundas: visões das independências no Piauí (1820-1840).

